# The influence of age-independent somatic driver alterations on clinical outcomes in paediatric and young adult thyroid cancer

**DOI:** 10.1530/ETJ-25-0310

**Published:** 2026-01-28

**Authors:** Sule Canberk, Amber Isaza, Mya Bojarsky, Mariana Simplício, Helena Barroca, Serra Z Akkoyunlu, Güven Günver, Isabel Almeida, Filippo Dello Iacovo, Anna M Carillo, Mariantonia Nacchio, Elena Vigliar, Claudio Bellevicine, Giancarlo Troncone, Riley Larkin, Ryan H Belcher, Vivian Weiss, Huiying Wang, Zubair Baloch, Fernando Schmitt, Andrew Bauer

**Affiliations:** ^1^ RISE-Health, Department of Pathology, Faculty of Medicine of The University of Porto, Porto, Portugal; ^2^ Division of Endocrinology and Diabetes, Children’s Hospital of Philadelphia, Philadelphia, Pennsylvania, USA; ^3^ Serviço de Anatomia Patológica, Centro Hospitalar Universitário de S João, Porto, Portugal; ^4^ Department of Biostatistics, Istanbul Faculty of Medicine, Istanbul University, Istanbul, Turkey; ^5^ Department of Public Health, University of Naples Federico II, Naples, Italy; ^6^ Pediatric Otolaryngology - Head and Neck Surgery at Vanderbilt Children’s Hospital, Nashville, Tennessee, USA; ^7^ Department of Pathology & Laboratory Medicine, University of Pennsylvania, Philadelphia, Pennsylvania, USA

**Keywords:** paediatric thyroid carcinoma, somatic driver alterations, RET and NTRK fusions, precision oncology, BRAF V600E, Paediatric age strata, paediatric age groups

## Abstract

**Background:**

Paediatric and young adult differentiated thyroid carcinoma (DTC) often presents at an advanced stage but carries an excellent prognosis. While age-related genomic differences from adult DTC are recognized, it remains unclear whether outcomes are driven by age or tumour biology.

**Methods:**

We analysed a multi-institutional cohort of 363 patients aged 0–25 years who underwent molecular testing and surgical management. Age was categorized using cut-offs at ≤8, 9–14, 15–18, and 19–25 years. The primary endpoint was disease status at last follow-up, categorized according to American Thyroid Association (ATA) response criteria. Multivariable ordered logistic regression was used to test the independent prognostic effect of somatic driver mutations while adjusting for age, sex, and follow-up duration.

**Results:**

Distinct age-related patterns of oncogenic drivers were observed: RET and NTRK1/3 fusions were predominant in younger patients, *BRAF* V600E was most frequent in adolescents, and *RAS* mutations were enriched in young adults. After adjustment, driver mutations independently predicted long-term outcomes. *NTRK1/3* fusions (aOR = 5.29, 95% CI: 1.77–15.79), *BRAF* V600E (aOR = 3.45, 95% CI: 1.37–8.70), and RET fusions (aOR = 3.34, 95% CI: 1.13–9.90) were associated with significantly higher odds of a non-excellent outcome. Conversely, *RAS* mutations showed a favourable trend, and all *DICER1*-mutant cases achieved excellent outcomes. While prognosis steadily improved with age, mutation status remained the dominant factor determining outcomes.

**Conclusion:**

Somatic drivers offer prognostic insights independent of age in paediatric and young adult DTC, establishing a molecular framework for precision risk stratification that complements traditional clinical staging and age-based assessments.

## Introduction

Differentiated thyroid carcinoma (DTC) in paediatric and young adult patients presents a unique clinical paradox. Compared to adults, paediatric DTC often shows a more aggressive initial presentation, with significantly higher rates of cervical lymph node involvement and distant metastasis at diagnosis (1). Despite this aggressive phenotype, the long-term prognosis is generally excellent with exceptionally low disease-specific mortality (2). The mismatch between clinical presentation and long-term outcomes challenges risk stratification and creates uncertainty about the appropriate intensity of initial treatment and follow-up, both of which carry substantial risks of morbidity in this young population.

Over the past decade, significant progress has been made in defining the molecular landscape of paediatric DTC, revealing a genomic profile that is markedly different from that of adult tumours (3). While adult DTC is dominated by point mutations, particularly *BRAF* V600E, paediatric disease is driven predominantly by kinase gene fusions, most commonly involving *RET* and *NTRK1/3* rearrangements (4, 5). The prevalence of these driver mutations is strongly age dependent: oncogenic fusions are most common in younger children, whereas the frequency of *BRAF* V600E and *RAS* point mutations increases progressively through adolescence and into young adulthood (6, 7, 8, 9, 10, 11).

While this molecular classification has improved the diagnostic accuracy of indeterminate thyroid nodules compared to cytologic and radiologic assessment alone, its utility for predicting long-term clinical outcomes after definitive surgery remains less clear (5, 12). The strong correlation between patient age and mutation type introduces a critical confounding variable. It is currently unknown whether the different clinical outcomes associated with certain alterations are attributable to the intrinsic biology conferred by the mutation itself or are simply a reflection of the patient’s age at diagnosis. Disentangling the independent prognostic impact of specific somatic drivers from the powerful influence of age is essential for developing a more precise, molecularly informed risk stratification framework that can facilitate personalized management (6, 9, 10).

In this study, we leveraged a multi-institutional cohort of paediatric and young adult patients with DTC to determine the independent association between canonical somatic driver alterations and disease status at last clinical follow-up. Using multivariable ordered logistic regression to adjust for age, sex, and follow-up duration, we tested the hypothesis that the underlying oncogenic driver is a primary determinant of clinical outcome. We demonstrate that tumours harbouring *RET* fusions, *NTRK1/3* fusions, and *BRAF* V600E mutations are independently associated with a significantly worse long-term prognosis, providing a new layer of molecular risk stratification that transcends patient age.

## Materials and methods

### Study design and patient cohort

This study was a retrospective analysis of a prospectively maintained, multi-institutional cohort database (REDCap) covering 2001–2024. The study population was drawn from four major international paediatric thyroid centres: Children’s Hospital of Philadelphia (*n* = 234; 63.4%), the University of Porto (*n* = 65; 17.6%), University of Naples Federico II (*n* = 54; 14.6%), and Vanderbilt University Medical Centre (*n* = 16; 4.3%).

We analysed a subset of project PEDIMAP patients aged 0–25 years with available somatic tumour molecular results. This yielded 363 patients for descriptive analyses of mutation prevalence and age distribution. A further 172 patients with complete data (mutation status, age, sex, follow-up, and ATA response) comprised the multivariable modelling cohort.

The Committees for the Protection of Human Subjects at the Children’s Hospital of Philadelphia (CHOP) reviewed the protocol Project PEDIMAP and determined it to be exempt on 29 April 2024 (CHOP IRB #23-021615; 45 CFR 46.104(d)(4)(iii)). A waiver of HIPAA authorization was granted per 45 CFR 164.512(i)(2)(ii) to access identifiable medical record data; data were analysed in de-identified form.

### Data collection and variable definitions

Clinical and demographic variables were abstracted from the database, including patient age at diagnosis, sex, and the duration of clinical follow-up, calculated in years from the date of surgery to the most recent clinical assessment. For analytical purposes, age at diagnosis was categorized at cut-offs 8, 14, 18, and 25 years into four strata: ≤8 (early childhood), 9–14 (early mid-adolescence), 15–18 (late adolescence), and 19–25 (young adulthood). This stratification was chosen to reflect key developmental periods and is consistent with prior studies that have established distinct, age-dependent molecular profiles in paediatric thyroid carcinoma, with kinase fusions predominating in younger children and the prevalence of *BRAF* V600E point mutations increasing significantly through adolescence (4, 6, 7, 8, 9, 10, 11, 12).

Pre-operative cytological diagnoses were classified according to the Bethesda System for Reporting Thyroid Cytopathology (3rd edition) (13). The histopathological follow-up was categorized as follows: benign, low-risk neoplasm, or malignant based on institutional pathology reports (14). Cases were not selected by histologic subtype; all surgically managed thyroid lesions undergoing somatic testing were included in the descriptive cohort. The primary study endpoint was the patient’s disease status at the last recorded clinical evaluation, which was defined according to the American Thyroid Association (ATA) guidelines for thyroid carcinoma (15). This outcome was structured as a four-level ordinal variable, ordered from most to least favourable: i) excellent response, defined as no biochemical or structural evidence of disease; ii) indeterminate response; iii) biochemically incomplete response; and iv) structurally incomplete response. For pre-specified sensitivity analyses, this variable was dichotomized into a binary outcome: excellent versus non-excellent (a composite category including indeterminate, biochemical incomplete, and structural incomplete responses).

### Somatic molecular profiling analysis and classification

Somatic mutation testing was conducted at each participating institution using either commercial or validated in-house next-generation sequencing (NGS) panels. These panels were designed to detect clinically relevant single nucleotide variants, small insertions/deletions (indels), and gene fusions in established thyroid carcinoma driver genes.

To facilitate a comprehensive analysis of driver-specific outcomes, patients were classified into one of seven mutually exclusive driver categories: *NTRK1/3* fusions, *BRAF* V600E, *RET* fusions, *RAS (NRAS/HRAS/KRAS*), *DICER1*, other canonical drivers (e.g. *ALK, PAX8-PPARG,* and *BRA*F non-V600E), and wild type (WT). Wild type was defined as tested and negative for all canonical drivers; ‘0/0 (0%)’ in descriptive tables indicates that testing was performed and no mutation-positive cases were observed in that age group. Cases with incomplete testing coverage were not assigned to WT. In cases with more than one alteration, patients were assigned to a single driver category according to a predefined hierarchy of canonical primary drivers (*RET/NTRK/ALK* fusions, *BRAF* V600E, *RAS, DICER1*, and others). Progression-associated alterations (e.g. *TERT* promoter, *TP53,* and *PTEN*) were not consistently assayed and were therefore not included in the driver classification.

### Statistical analysis

Cohort characteristics were summarized with descriptive statistics. Associations among categorical variables (e.g. mutation group by age band) were tested with Pearson’s chi-squared test; Fisher’s exact (Fisher–Freeman–Halton for r × c tables) was used when expected cell counts were <5. All tests were two-sided with *α* = 0.05. The primary analysis used a proportional-odds (cumulative logit) model for the four-level outcome at last evaluation (excellent, indeterminate, biochemical incomplete, and structural incomplete). The main predictor was molecular driver group; models were adjusted for age group (≤8, 9–14, 15–18, and 19–25 years), sex, and follow-up duration (years). The observed outcome distributions by age × mutation group were summarized in descriptive heatmaps. Information on adjuvant therapy (radioactive iodine administration/dose, TSH suppression, external beam radiation, or targeted kinase inhibitors such as RET/TRK inhibitors) was not uniformly available in the registry. RAI status (yes/no) was recorded in the complete case modelling cohort (*n* = 172), and a sensitivity analysis additionally adjusted for RAI exposure. Thus, treatment modality was not modelled in the primary analysis, and residual confounding by therapy remains possible.

Reference levels were wild type (driver), 15–18 years (age), and female (sex). Results are reported as adjusted proportional odds ratios (aORs) with 95% CIs and Wald *P*-values. To aid clinical interpretation, we computed model-based predicted probabilities for each outcome from the fitted proportional-odds model, first for a reference profile (female, 15–18 years, median follow-up) and then by age group (no outcome dichotomization). A pre-specified sensitivity analysis dichotomized the outcome (non-excellent vs excellent) and used Firth’s penalized logistic regression to address complete separation observed in the DICER1 group; the corresponding unpenalized maximum-likelihood results are presented in the supplementary material. We also conducted a sensitivity analysis additionally adjusting for RAI exposure (yes/no) within the complete-case cohort (*n* = 172), to assess potential confounding by treatment modality. Age-stratified predicted probabilities were computed from the proportional-odds model. For age × mutation cells with zero observed cases (e.g. *BRAF* V600E at ≤8 years), predictions represent extrapolation from fitted coefficients informed by other strata and covariates; no additional regularization was applied for these cells.

## Results

### Cohort characteristics and age-dependent distribution of somatic mutations

The study cohort comprised 363 paediatric and young adult patients (0–25 years) who underwent thyroid surgery and had available somatic testing data. The patient population was predominantly adolescents and young adults, with 151 patients (41.6%) in the 15–18-year age group and 101 patients (27.8%) in the 19–25-year group. Among the age-known tested cohort (*n* = 363), follow-up was available for 214 patients (mean 3.52 years; range 0.01–12.98). In the complete-case modelling set (*n* = 172), the mean follow-up was 4.04 years (range 0.13–12.98).

**Figure 1:**
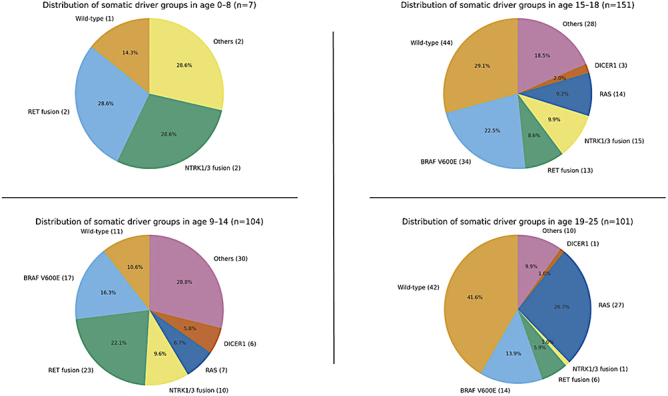
Association between somatic mutation status and age group (*n* = 363). *Data are *n* (%). Cohort restricted to somatically tested patients with a known age group (*n* = 363). Percentages in positive/negative rows are row percentages; the total row shows column percentages (age distribution). Overall *P*-value: Pearson *χ*^2^ (2 × 4); when expected counts were <5, the Fisher–Freeman–Halton exact test was used as a sensitivity check (results unchanged). *NTRK*1/3 =* NTRK1* or *NTRK3* fusion (union). *RAS* (N/H/K) = *NRAS/KRAS/HRAS.* Others = *ALK, PAX8–PPARG, BRAF *non-V600E, and other specified canonical alterations. Negative indicates the absence of the specified alteration and may include tumours with other driver mutations as well as wild type. Wild type denotes cases tested negative for all specified canonical drivers (Supplemental Table S1).

A primary descriptive finding was that oncogenic drivers clustered within distinct age strata (Fig. 1; Supplemental Table S1 (see section on Supplementary materials given at the end of the article). *RET* and *NTRK1/3* fusions were concentrated in children under 15 years, whereas *BRAF* V600E was largely confined to the 15–18-year band and R*AS* mutations were enriched in young adults (19–25 years). Within-age-group driver distributions are summarized in Fig. 1: *NTRK1/3* fusions contributed a relatively larger share of somatic drivers in the 9–14- and 15–18-year groups, although absolute numbers remained modest in the youngest age band (0–8 years; *n* = 7). *BRAF* V600E mutations were absent in early childhood (0% in the 0–8-year group) and increased in prevalence with age, peaking in the 15–18-year group (52.3% of all *BRAF* V600E-positive cases). In contrast, *RAS* mutations were predominantly identified in young adults, with 56.3% of all *RAS*-mutant cases occurring in the 19–25-year age group (*P* < 0.001). Because the ≤8-year group contained only seven patients, age-band percentages in that stratum should be interpreted descriptively.

**Figure 2:**
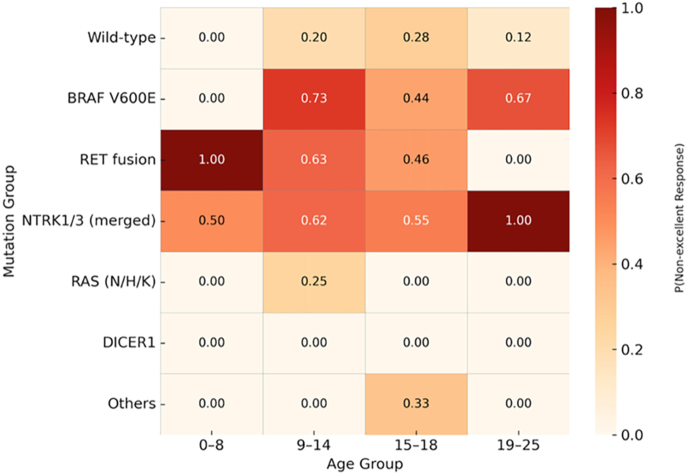
Observed probability of non-excellent response by age group and mutation group (tested-only cohort). Heatmap showing observed frequencies of a non-excellent response (indeterminate, biochemical incomplete, or structural incomplete ATA outcome) across age strata (0–8, 9–14, 15–18, and 19–25 years) and mutually exclusive mutation categories. The values represent proportions of non-excellent response within each mutation–age cell. ‘0/0 (0%)’ indicates that no mutation-positive cases were observed in that stratum; testing was performed, and the mutation was absent (see Supplemental Table S2). The darker shading (red) indicates a higher probability of non-excellent response; the lighter shading indicates a lower probability.

To complement these prevalence patterns, the observed outcome distributions by age × mutation group are shown as a heatmap (Fig. 2) and detailed in Supplemental Table S2. For example, while no *BRAF* V600E cases were detected in ≤8 years, *RET* and *NTRK1/3* fusions in this youngest group were frequently associated with non-excellent outcomes, whereas *RAS* mutations, predominantly in the 19–25-year group, showed consistently favourable observed responses.

Histologically, the cohort comprised predominantly PTC (*n* = 232), with smaller numbers of FTC (*n* = 18), IEFV-PTC (*n* = 5), and a heterogeneous pooled category of other histologies (*n* = 108), which includes rare malignant entities and benign/low-risk diagnoses (Supplemental Tables S3 and S12). As shown in Supplemental Table S3, PTC and IEFV-PTC were enriched for *BRAF* V600E, *RET*, and *NTRK1/3* fusions, whereas FTC more often harboured *RAS* and *DICER1* alterations.

RAI use varied across both age and driver categories (Supplemental Table S4). All patients aged 0–8 years received RAI (7/7), whereas 56.9–72.4% of older age groups were treated. By somatic driver, RAI was most frequently administered to *RET* fusion-positive tumours (82.1%), with similar use for *BRAF* V600E, *NTRK1/3* fusions, wild type, and ‘others’ driver groups (∼64–68%). RAI use was less frequent in *DICER1*-mutant tumours (44.4%) and rare in *RAS*-positive tumours (6.7%).

### Somatic driver mutations are independent predictors of disease status

After adjusting for age, sex, and duration of follow-up in the ordered logistic regression model, somatic mutation status emerged as a powerful and independent predictor of clinical outcome at last follow-up (Table 1). Patients harbouring tumours with *NTRK1/3* fusions had the highest odds of a worse disease status compared to their wild-type counterparts (aOR = 5.29; 95% CI: 1.77–15.79; *P* = 0.003). Similarly, both *BRAF* V600E mutations (aOR = 3.45; 95% CI: 1.37–8.70; *P* = 0.009) and *RET* fusions (aOR = 3.34; 95% CI: 1.13–9.90; *P* = 0.029) were independently associated with significantly worse clinical outcomes. In contrast, *RAS* mutations were not significantly associated with outcome, showing a trend toward a more favourable prognosis (aOR = 0.39; 95% CI: 0.04–3.61; *P* = 0.408). All patients with *DICER1* mutations had excellent outcomes, leading to complete separation in the model (aOR → 0), which precluded a finite estimate but strongly suggested a favourable prognosis. ‘Others’ showed no significant association with outcome.

**Table 1:** Adjusted proportional odds ratios for worse disease status at last clinical evaluation (ordered logistic regression; *n* = 172).

Mutation group	aOR	95% CI	*P*-value
*BRAF* V600E	3.45	1.37–8.70	**0.009**
*RET fusion*	3.34	1.13–9.90	**0.029**
*NTRK1/3*	5.29	1.77–15.79	**0.003**
*RAS (N/H/K)*	0.39	0.04–3.61	0.408
*DICER1*	∼0*	-	0.984
Others	1.96	0.64–6.02	0.237

* Indicates separation; no worse outcomes.

As treatment variables, such as RAI administration, were not included in the main models, these associations represent age- and sex-adjusted mutation effects within real-world treatment patterns. A sensitivity model additionally adjusting for RAI exposure is provided in the supplementary material, which showed consistent mutation effects (Supplemental Table S5).

RAI use differed by age and driver group (Supplemental Table S4), and sensitivity analyses adjusting for RAI showed consistent mutation effects (Supplemental Table S5).

### Clinical translation: predicted outcome probabilities by mutation status

**Figure 3:**
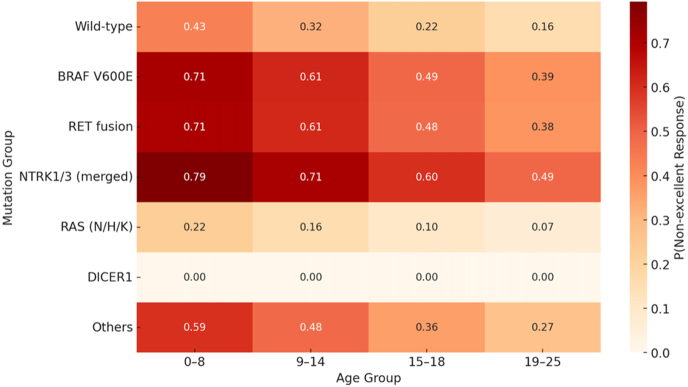
Predicted probability of non-excellent outcome by age group and mutation group. Predicted probabilities of a non-excellent outcome (indeterminate, biochemical incomplete, or structural incomplete) from the ordered logistic regression model including mutation group and age group, adjusted for sex and follow-up duration. Age-banded predictions show the highest predicted risk for NTRK1/3 and RET across age bands, intermediate risk for BRAF V600E, the lowest for RAS, and uniformly excellent outcomes for DICER1 (complete separation). Treatment effects were not included in the primary model; probabilities reflect outcomes under real-world care in this cohort. Predictions for strata with no observed mutation-positive cases (e.g. BRAF V600E at ≤8 years) reflect model extrapolation and should be interpreted with caution. The darker shading indicates a higher predicted probability of a non-excellent outcome.

To translate these adjusted odds ratios into more clinically accessible metrics, model-based predicted probabilities for each of the four outcome categories were calculated for a reference patient profile (female, 15–18 years old, median follow-up time) (Table 2; Fig. 3). This analysis revealed substantial differences in expected clinical course based on the underlying driver mutation.

**Table 2:** Model-based predicted probabilities of clinical outcomes by mutation group for a reference profile.

Mutation group	*P* (excellent)	*P* (indeterminate)	*P* (biochemical)	*P* (structural)
Wild type	0.787	0.053	0.108	0.052
*BRAF* V600E	0.517	0.086	0.238	0.159
*RET fusion*	0.525	0.085	0.235	0.155
*NTRK1/3*	0.411	0.086	0.278	0.224
*RAS (N/H/K)*	0.904	0.026	0.049	0.021
*DICER1*	∼1.000	∼0.000	∼0.000	∼0.000
Others	0.653	0.074	0.176	0.097

The probability of achieving an excellent response was lowest for patients with *NTRK1/3* fusions (41.1%). Meanwhile, patients with *RAS* mutations had a high probability of an excellent response (90.4%), as did those with wild-type tumours (78.7%). Conversely, the probability of experiencing the worst outcome, structural incomplete response, was highest for the *NTRK1/3* group at 22.4%, compared to only 5.2% for the wild-type group and 2.1% for the *RAS*-mutant group. Patients with *BRAF* V600E and *RET* fusions demonstrated intermediate prognoses, with predicted probabilities of an excellent response of 51.7 and 52.5%, respectively. 

These findings demonstrate that the genetic driver defines a quantitative spectrum of biological behaviour. The model effectively stratifies patients into distinct prognostic tiers: a very high-risk tier (*NTRK1/3*), a high-risk tier (*BRAF* V600E, *RET*), a favourable-risk tier (wild type), and a very favourable-risk tier (*RAS*, *DICER1*). For a typical adolescent female patient, an *NTRK1/3* fusion confers less than a 50% chance of being disease-free and approximately a one-in-four chance of having persistent structural disease, providing a clear framework for risk-stratified clinical management (Table 2; Fig. 3).

### Age-stratified predictions

The combined influence of age and mutation status on the probability of a non-excellent outcome was visualized by plotting model-based, sex- and follow-up-adjusted predicted probabilities for each mutation group across all four age bands (Fig. 3). This visualization confirmed that the prognostic disadvantage conferred by *NTRK1/3* and *RET* fusions was evident across all age groups, with these drivers consistently associated with the highest probability of a non-excellent outcome.

For most mutation types, the prognosis appeared to improve with increasing patient age. For instance, the predicted risk of a non-excellent outcome decreased from ∼71% in the ≤8-year group to ∼39% in the 19–25-year group (Supplemental Table S6). The favourable prognosis associated with *RAS* and *DICER1* mutations was also consistent across all age groups, with their predicted risks of a non-excellent outcome remaining low throughout the entire age spectrum. In the ≤8-year age band, the fitted model assigned the highest predicted probability of a non-excellent outcome to *BRAF* V600E (71.4%). This estimate should be interpreted with caution because no *BRAF* V600E-positive cases were observed in this stratum (0/0 in Supplemental Table S2); thus, the extreme value reflects model extrapolation from sparse data rather than an observed biological signal. These probabilities are model based and conditional on age, sex, and follow-up time. Because treatment (e.g. RAI administration) was not included in the main models, these results should be interpreted as associations under real-world care patterns rather than therapy-adjusted (biology-only) effects. A sensitivity model analysis including RAI (yes/no) was performed in the complete-case cohort (*n* = 172) and confirmed that the relative mutation effects were unchanged (Supplemental Table S5).

### Sensitivity analysis: Firth’s logistic regression of the binary outcome

Because the primary analysis used an ordinal outcome with a proportional-odds link, we evaluated whether the results depended on that specification. As a pre-specified sensitivity check, we modelled a binary endpoint (non-excellent vs excellent) using Firth’s penalized logistic regression, which addresses sparse data and complete separation (notably in the DICER1 group). The direction and magnitude of effects matched the primary model (Table 3). After adjustment for age group, sex, and follow-up time, *BRAF* V600E (aOR = 2.69; 95% CI: 1.20–6.03; *P* = 0.016), *RET* fusions (aOR = 2.64; 95% CI: 1.13–6.14; *P* = 0.025), and *NTRK1/3* fusions (aOR = 2.65; 95% CI: 1.07–6.58; *P* = 0.036) were associated with higher odds of a non-excellent outcome compared with wild type, whereas *RAS* was not significant (aOR = 0.54; 95% CI: 0.14–2.06; *P* = 0.366). Unpenalized estimates and model diagnostics are provided in Supplemental Table S7. A supplementary model additionally adjusting for RAI exposure yielded consistent results, underlying robustness across model specifications.

**Table 3:** Penalized logistic regression for non-excellent vs excellent outcome (*n* = 172).

Mutation group	aOR*	95% CI	*P*-value
*BRAF* V600E	2.69	1.20–6.03	0.016
*RET fusion*	2.64	1.13–6.14	0.025
*NTRK1/3 *(merged)	2.65	1.07–6.58	0.036
*RAS (N/H/K)*	0.54	0.14–2.06	0.366
*DICER1*	0.43	0.09–1.96	0.273
Others	0.78	0.27–2.27	0.647

* Non-excellent.

### Ancillary descriptive findings

Ancillary analyses showed a clear improvement in outcome with increasing age (Supplemental Table S8), but no meaningful differences across broad histopathology groupings as benign, low-risk neoplasm and malignant (Supplemental Table S9) and no consistent discrimination by Bethesda category (Supplemental Table S10). Unadjusted mutation effects and age-stratified predictions (Supplemental Tables S2, S6, and S11) mirrored the adjusted models, and an unpenalized sensitivity model yielded similar directions (Supplemental Table S7). In addition, seven patients had a final benign diagnosis (including follicular adenoma and -follicular nodular disease) (Supplemental Table S12). All were older than 8 years and had no biochemical or structural evidence of disease at last follow-up; these benign cases were not included in the multivariable ATA outcome modelling. Their molecular profiles comprised DICER1 (*n* = 2), ‘others’ drivers (*n* = 3), and wild-type tumours (*n* = 2).

## Discussion

This multi-institutional study represents one of the largest integrated analyses to date of somatic driver alterations in paediatric and young adult thyroid carcinoma with ATA-based outcome assessment (15). By combining genomic, clinicopathologic, and outcome data from four international centres, we demonstrate that the identity of the oncogenic driver exerts a powerful and age-independent influence on disease behaviour. Both descriptive and model-based analyses converge on a consistent finding: kinase fusions, particularly those involving *NTRK1/3* and *RET*, delineate the most aggressive disease spectrum, whereas *RAS* and *DICER1* mutations define a biologically indolent, low-risk end of the continuum (Tables 1, 2, 3; Figs 1, 2, 3; Supplemental Tables S1, S2, S3, S4, S5, S6, S7, and S11). *BRAF* V600E occupies an intermediate position, its prevalence rises sharply in adolescence and early adulthood, and its risk profile parallels that of adult-type papillary thyroid carcinoma but remains less adverse than that of fusion-positive tumours in this younger population (Tables 1, 2, 3; Figs 1, 2, 3).

A central challenge in paediatric thyroid oncology has been to reconcile the aggressive clinical presentation, characterized by high rates of nodal and distant metastasis, with the excellent overall long-term survival (3, 10, 12). Our study addresses a key aspect of this paradox by disentangling the prognostic roles of tumour biology (the alterations) and host factors (patient age). The descriptive analysis confirmed the strong association between age and mutation type: *RET* and *NTRK* fusions predominated in younger children, whereas *BRAF* V600E clustered in mid-to-late adolescence, and *RAS* mutations were most common in young adults. These findings are consistent with multiple independent series that stratified paediatric cohorts into discrete age bands (4, 6, 8, 9, 10, 16). In the Belarusian and Ukrainian post-Chernobyl cohorts, for example, *RET/PTC* fusions were overwhelmingly enriched in children under 15 years of age, particularly those diagnosed before adolescence, while *BRAF* V600E was rare in this group and only emerged in patients older than 15 (17, 18). Similarly, Galuppini *et al.* analysed 59 paediatric patients and showed that children under 15 years presented with a more extensive disease and higher rates of nodal and distant metastases and were more likely to harbour *RET/PTC* fusions, whereas *BRAF* V600E mutations (16% overall) were concentrated in adolescents ≥15 years (19). In a large Korean series, Lee *et al.* confirmed that fusion oncogenes were found predominantly in patients under 10 years of age, while *BRAF* V600E was detected mostly in adolescents, with the median age of fusion-positive patients being significantly lower than that of *BRAF*-positive cases (6). Our cohort aligned these patterns across four clinically relevant strata (≤8, 9–14, 15–18, and 19–25 years), highlighting that while mutation type strongly segregates by age, the prognostic effect of the mutation itself persists independently of age. Together, these studies and our own data are consistent with the interpretation that the developing thyroid epithelium is particularly susceptible to fusion-generating genomic injury, whether of endogenous or environmental origin.

Beyond their distribution, the clinical implications of these molecular classes are clear in our adjusted outcome modelling. Tumours with *NTRK1/3* or *RET* fusions had the highest odds of a worse ATA response (incomplete response), three to five times that of wild-type counterparts, after adjusting for age, sex, and follow-up duration (Table 1; Fig. 3). The observed probabilities of a non-excellent outcome exceeded 60% for most fusion-positive groups, in contrast to 20–30% for wild-type tumours and <10% for *RAS*-mutant tumours (Supplemental Table S2; Fig. 2). These results closely mirror the findings of Franco *et al.*, who showed that fusion-positive carcinomas exhibit higher rates of lymph node and distant metastases, extrathyroidal extension, and persistent disease, even after controlling for stage and age (16). Our results extend these findings by showing that the adverse behaviour of fusions remains evident when modelled against confounding factors, pointing the driver mutation itself as a dominant biological determinant of outcome. To translate these adjusted odds ratios into clinically actionable metrics, our model-based predicted probabilities demonstrate that for a reference patient profile (female, 15–18 years, median follow-up), *NTRK1/3* fusion-positive tumours have only a 41.1% probability of achieving excellent response with a 22.4% risk of structural incomplete response, whereas *RAS*-mutant tumours show 90.4% probability of excellent response with only 2.1% risk of persistent structural disease (Table 2). These quantitative estimates enable clinicians to counsel patients with molecular-based precision and tailor surveillance and treatment intensity accordingly (20).

Integration of radioactive iodine (RAI) exposure further contextualizes these associations. In our cohort, RAI utilization differed markedly by driver, being highest among *RET*-positive tumours and lowest among *RAS*-mutant tumours (Supplemental Table S4). When RAI was included as a covariate in sensitivity modelling, the driver–outcome relationships remained stable, while RAI therapy itself was independently associated with lower odds of a non-excellent outcome (aOR = 0.32, 95% CI: 0.14–0.77) (Supplemental Table S5). This supports that while treatment contributes to outcome variability, molecular biology exerts the predominant influence. The reduced responsiveness of *RET*- and *NTRK*-driven carcinomas to RAI reflects well-characterized mechanisms: downstream MAPK and PI3K pathway activation leading to repression of sodium–iodide symporter (NIS) expression and functional iodine uptake loss (9, 21). These biologic features also explain the impressive therapeutic sensitivity of these same tumours to selective *RET* and TRK inhibitors (22, 23), which can restore NIS expression and iodine avidity in some cases.

The behaviour of *BRAF* V600E in this age spectrum warrants particular attention. In our cohort, *BRAF* V600E was absent in early childhood and increased through adolescence (Fig. 1; Supplemental Table S1). In the adjusted proportional-odds model, *BRAF* V600E was associated with higher odds of a worse ATA response than wild type/*RAS*, but lower risk than *RET* or *NTRK* fusions, supporting an intermediate-risk phenotype (Tables 1 and 2; Fig. 3). These findings align with paediatric series showing that *BRAF* V600E is not an independent high-risk driver once age, stage, and histotype are considered (8, 24, 25). The modelled age gradient (the predicted probability of a non-excellent outcome decreased from ∼71% at ≤8 years to ∼38% at 19–25 years) should be interpreted in light of small cell sizes at the age extremes for *BRAF*, and the ≤8-year estimate reflects extrapolation because no *BRAF* V600E cases were observed in that band (observed counts – ≤8: *n* = 0; 9–14: *n* = 11; 15–18: *n* = 18; 19–25: *n* = 3; Supplemental Table S2). Overall, the data indicate that *BRAF*-driven tumours in young patients are intermediate in risk, worse than wild type/*RAS* but less adverse than fusion-positive disease, consistent with a developmental context in which differentiation programs may be more preserved than in adults. Franco *et al.* emphasized that BRAF’s prognostic significance in children differs from adults, reflecting context-dependent effects (16).

Conversely, *RAS* and *DICER1* mutations define a distinctly favourable phenotype. All D*ICER1*-mutant tumours in our series achieved excellent ATA responses, and *RAS*-mutant cases exhibited the lowest rates of persistence across age groups. This behaviour aligns with reports that *RAS*-mutant and *DICER1*-associated neoplasms often represent encapsulated, follicular-patterned lesions with minimal invasion (26, 27). Their molecular signalling preserves differentiation programs, reflected in maintained iodine avidity and indolent course. It is important to note, however, that while our cohort showed uniformly favourable *DICER1* outcomes, approximately 5% of *DICER1*-mutant thyroid tumours in the literature exhibit high-grade features, including poorly DTC and thyroblastoma, warranting continued surveillance despite generally favourable biology (28, 29). These results reinforce that driver identity encapsulates both lineage commitment and biological behaviour, providing a mechanistic basis for risk diversity in paediatric thyroid tumours.

An important ancillary finding was the absence of prognostic discrimination by either the Bethesda cytology category (Supplemental Table S10, *χ*^2^ = 18.08, *P* = 0.164) or the broad histopathological classification (Supplemental Table S9, *χ*^2^ = 4.64, *P* = 0.665). This observation is mechanistically consistent with the paediatric context: the Bethesda system was designed and optimized for adult thyroid nodules, where the spectrum of lesions and cytomorphologic features differs fundamentally. In paediatric patients, fusion-driven tumours may exhibit deceptively bland cytology lacking classic papillary nuclear features, while malignancy rates in nodules are substantially higher than in adults. These findings show that long-term outcome prediction in paediatric thyroid carcinoma depends more on molecular driver identity than on the cytologic or broad histopathological classification, supporting molecularly integrated diagnostic approaches.

Clinically, these data provide a robust argument for molecularly informed risk stratification in paediatric thyroid carcinoma. The presence of an *RET* or *NTRK* fusion identifies patients who likely benefit from more complete surgery (thyroidectomy rather than hemithyroidectomy), as well as potential eligibility for selective kinase inhibitors in widely invasive and/or RAI refractory cases. Conversely, the detection of *RAS* or D*ICER1* alterations supports a conservative management approach (lobectomy rather than thyroidectomy without central neck lymph node dissection) (30). The probabilistic framework used here translates molecular findings into quantitative outcome estimates, facilitating integration of genomics into ATA-based paediatric risk categories and, potentially, stratification of clinical care.

Several limitations must be acknowledged. Although RAI exposure data were available for all modelled cases, additional therapeutic details, such as dose intensity, surgical extent, and TSH suppression, were not uniformly captured and could not be incorporated into regression models. Nevertheless, a sensitivity analysis including RAI (yes/no) in the complete-case cohort (*n* = 172) confirmed that the driver–outcome relationships were stable. Molecular testing was also not performed on a single harmonized platform across centres, and this has direct implications for the apparent ‘wild-type/driver-negative’ fraction. Published paediatric thyroid carcinoma series demonstrate that ‘driver-negative’ rates are strongly methodology dependent: limited non-NGS or DNA-only approaches can leave >50% of tumours without an identifiable driver, whereas comprehensive RNA-inclusive profiling (DNA + RNA panels or transcriptome sequencing) reduces the unexplained fraction to low single digits (approximately 2–7%), consistent with the fusion-driven biology of paediatric papillary thyroid carcinoma. Consequently, a negative result on a DNA-only panel is insufficient to exclude clinically relevant oncogenic drivers, particularly RET and NTRK fusions, which dominate the paediatric landscape and are clinically actionable. In our multi-institutional cohort, ‘wild type’ was defined operationally as ‘no canonical driver detected on the local testing platform’ and comprised 27.0% of the full tested cohort (98/363). In the subsets with ATA disease status available for the age × driver outcome heatmap (Table S2; *n* = 187), wild type comprised 40.6% (76/187). These values therefore fall within the published range and are most appropriately interpreted as reflecting real-world platform heterogeneity and incomplete fusion capture rather than diagnostic misclassification (31, 32, 33, 34). The median follow-up of 4 years is appropriate for paediatric thyroid carcinoma but may under-ascertain very late recurrences. The apparent high risk assigned to *BRAF* V600E in the ≤8-year age band is a model artefact: we observed no *BRAF* V600E-positive tumours in this band (Supplemental Table S2), consistent with the observed heatmap (Fig. 2), yet the model produced an extreme estimate for that cell (Supplemental Table S6). This reflects extrapolation from sparse data rather than an observed biological effect and should not be interpreted as evidence that *BRAF* V600E is especially adverse in early childhood. Multiple large studies have reported a *BRAF* V600E prevalence of ∼0–1% in children <10 years, with fusion oncogenes (*RET/PTC, NTRK,* and *ALK*) predominating as molecular drivers of aggressive disease in this age group. Finally, certain rare drivers (e.g. *ALK* and *BRAF fusion* and *PAX8–PPARG*) were underrepresented, limiting precision and precluding detailed analyses. In addition, histologic subtype was abstracted from local institutional pathology reports without central review and spans multiple editions of the WHO classification of thyroid neoplasms, which may attenuate any independent effect of detailed histotype in this retrospective cohort. We also did not perform a central histopathological re-review; histologic diagnoses for *RAS*-mutant tumours reflect local institutional reporting over multiple WHO eras, so some lesions classified as carcinoma in this dataset might today be reclassified as NIFTP or other low-risk entities. Within this context, the favourable RAS-mutant outcomes are likely influenced by an enrichment for NIFTP and biologically indolent encapsulated variants. However, this aligns with evidence that *RAS*-driven tumours, even those meeting carcinoma criteria, typically exhibit an indolent course distinct from fusion-driven disease, supporting conservative management. Despite these constraints, the strengths of this study include its multi-institutional scope, uniform outcome coding, comprehensive molecular annotation, and what is, to our knowledge, the largest harmonized modelling cohort reported with proportional-odds analyses in this context.

In conclusion, this study demonstrates that somatic driver alterations serve as robust, age-independent prognostic markers in paediatric and young adult DTC. By mapping both their age distribution and prognostic impact, we propose a risk framework: *RET* and *NTRK* fusions, enriched in younger patients, define the highest risk tier; *BRAF* V600E, emerging in adolescence, marks an intermediate-risk tier; and *RAS* and *DICER1*, clustering in late adolescence and young adulthood, define favourable tiers. This model resolves the ambiguity between age and tumour biology and aligns with the evolving paradigm of precision oncology in thyroid carcinoma where treatment intensity can be matched not only to tumour stage but to the molecular and age context of the patient.

## Supplementary materials



## Declaration of interest

The authors declare that there is no conflict of interest that could be perceived as prejudicing the impartiality of the research reported.

## Funding

This work was financed by the European Regional Development Fund (ERDF), through the North Regional Operational Program in the framework of the project MitOConexão: Investigating the Mitochondrial Nexus in Onco-Cardiology (Reference NORTE2030-FEDER-02713500).

## Author contribution statement

SC and AJB conceived and designed the study. AI and MB collected the data and accrued the REDCap case. AI and MB coordinated the study and were involved in contributor navigation. SC, AI, MB, MS, HB, SZA, GG, IA, FDI, AMC, MN, EV, CB, GT, RL, RHB, VW, HW, ZB, FS, and AB were involved in data provision and institutional case contribution. SC drafted the manuscript. SZA, GG, and SC performed statistical analyses. SC, AI, MB, MS, HB, SZA, GG, IA, FDI, AMC, MN, EV, CB, GT, RL, RHB, VW, HW, ZB, FS, and AJB critically reviewed and approved the manuscript.
