# Supplementary material for: The influence of age-independent somatic driver alterations on clinical outcomes in paediatric and young adult thyroid cancer
Source: Eur Thyroid J. 2026 Jan 28;15(1):ETJ250310. doi: 10.1530/ETJ-25-0310 (PMC13138597; doi:10.1530/ETJ-25-0310)
Supplement: Supplementary file 1 [file supplementary_materials.pdf]

Table S1. Association between Somatic Mutation Status and Age Group (N=363)

Mutation Group	0-8 years	9-14 years	15-18 years	19-25 years	Overall p-value*
***BRAF* V600E**					
BRAF V600E Positive	0 (0.0%)	17 (26.2%)	34 (52.3%)	14 (21.5%)	0.171
BRAF V600E Negative	7 (2.3%)	87 (29.2%)	117 (39.3%)	87 (29.2%)	
Total	7 (1.9%)	104 (28.7%)	151 (41.6%)	101 (27.8%)	
NTRK* fusion *(1/3* combined*)					
NTRK1/3 fusion Positive	2 (7.1%)	10 (35.7%)	15 (53.6%)	1 (3.6%)	0.0065
NTRK1/3 fusion Negative	5 (1.5%)	94 (28.1%)	136 (40.6%)	100 (29.9%)	
Total	7 (1.9%)	104 (28.7%)	151 (41.6%)	101 (27.8%)	
***RET* fusion**					
RET fusion Positive	2 (4.5%)	23 (52.3%)	13 (29.5%)	6 (13.6%)	< 0.001
RET fusion Negative	5 (1.6%)	81 (25.4%)	138 (43.3%)	95 (29.8%)	
Total	7 (1.9%)	104 (28.7%)	151 (41.6%)	101 (27.8%)	
***RAS (N/H/K* combined)**					
RAS Positive	0 (0.0%)	7 (14.6%)	14 (29.2%)	27 (56.3%)	< 0.001
RAS Negative	7 (2.2%)	97 (30.3%)	137 (42.8%)	74 (23.1%)	
Total	7 (1.9%)	104 (28.7%)	151 (41.6%)	101 (27.8%)	
DICER1					
DICER1 Positive	0 (0.0%)	6 (60.0%)	3 (30.0%)	1 (10.0%)	0.156
DICER1 Negative	7 (2.0%)	98 (27.8%)	148 (41.9%)	100 (28.3%)	
Total	7 (1.9%)	104 (28.7%)	151 (41.6%)	101 (27.8%)	
**Others**					
Others Positive	2 (2.7%)	30 (40.5%)	31 (41.9%)	11 (14.9%)	0.0148
Others Negative	5 (1.7%)	74 (25.6%)	120 (41.5%)	90 (31.1%)	
Total	7 (1.9%)	104 (28.7%)	151 (41.6%)	101 (27.8%)	

***** Data are n (%). Cohort restricted to somatically tested patients with a known age group (N=363). Percentages in Positive/Negative rows are row percentages; the Total row shows column percentages (age distribution). Overall p-value: Pearson χ^2 (2×4); when expected counts were <5, a Fisher–Freeman-Halton exact test was used as a sensitivity check (results unchanged). *NTRK1/3 = NTRK1 or NTRK3* fusion (union). *RAS (N/H/K) = NRAS/KRAS/HRAS*. Others = *ALK, PAX8–PPARG, BRAF non*-V600E, and other specified canonical alterations. Negative' indicates absence of the specified alteration and may include tumours with other driver mutations as well as wild-type. Wild-type denotes cases tested and negative for all specified canonical drivers.

Table S2. Observed Probability of Non-excellent Response by Mutation Group and Age (Tested-only cohort)

Non-excellent includes Biochemical Incomplete, Structural Incomplete, and Indeterminate response.

Mutation group	0–8	9–14	15–18	19–25
**Wild-type**	0/1 (0.0%)	3/15 (20.0%)	10/36 (27.8%)	3/24 (12.5%)
**BRAF V600E**	0/0 (0.0%)	8/11 (72.7%)	8/18 (44.4%)	2/3 (66.7%)
**RET fusion**	1/1 (100.0%)	12/19 (63.2%)	5/11 (45.5%)	0/2 (0.0%)
**NTRK1/3 (merged)**	1/2 (50.0%)	5/8 (62.5%)	6/11 (54.5%)	1/1 (100.0%)
**RAS (N/H/K)**	0/0 (0.0%)	1/4 (25.0%)	0/5 (0.0%)	0/0 (0.0%)
**DICER1**	0/0 (0.0%)	0/3 (0.0%)	0/0 (0.0%)	0/1 (0.0%)
**Others**	0/0 (0.0%)	0/7 (0.0%)	1/3 (33.3%)	0/1 (0.0%)

0/0 (0%)” indicates no mutation-positive cases observed in that age stratum; testing was performed and the mutation was absent.

Table S3. Distribution of histologic subtypes by somatic driver group (tested cohort).

Histology	Wild-type	BRAF V600E	RET fusion	NTRK1/3 fusion	RAS	DICER1	Others (forced = 74)	Row_N
**PTC**	46 (19.8%)	58 (25.0%)	40 (17.2%)	28 (12.1%)	7 (3.0%)	5 (2.2%)	48 (20.7%)	232
**FTC**	7 (38.9%)	0 (0.0%)	0 (0.0%)	0 (0.0%)	4 (22.2%)	3 (16.7%)	4 (22.2%)	18
**IEFV-PTC**	5 (100.0%)	0 (0.0%)	0 (0.0%)	0 (0.0%)	0 (0.0%)	0 (0.0%)	0 (0.0%)	5
**Other histologies**	40 (37.0%)	7 (6.5%)	4 (3.7%)	0 (0.0%)	33 (30.6%)	2 (1.9%)	22 (20.4%)	108
**Total (tested cohort)**	98 (27.0%)	65 (17.9%)	44 (12.1%)	28 (7.7%)	44 (12.1%)	10 (2.8%)	74 (20.4%)	363

Table S4 – Radioactive iodine (RAI) use by age group and by driver group

**Age group**	**RAI No**	**RAI Yes**	**Total with known RAI**	**% receiving RAI**
**0–8**	0	7	7	100.0%
**9–14**	27	71	98	72.4%
**15–18**	56	74	130	56.9%
**19–25**	13	25	38	65.8%
**Driver group**	**RAI No**	**RAI Yes**	**Total with known RAI**	**% receiving RAI**
**Wild-type**	21	44	65	67.7%
***BRAF* V600E**	18	38	56	67.9%
***RET* fusion**	7	32	39	82.1%
NTRK1/3	9	19	28	67.9%
RAS (N/H/K)	14	1	15	6.7%
DICER1	5	4	9	44.4%
**Others**	22	39	61	63.9%

Table S5. Sensitivity analysis including RAI (complete-case cohort, n=172)

Mutation group	aOR (non-excellent)	95% CI	p-value
***BRAF* V600E**	3.90	1.37–11.10	0.011
***RET* fusion**	4.06	1.38–11.94	0.011
NTRK1/3	3.63	1.14–11.55	0.029
RAS (N/H/K)	0.17	0.02–1.74	0.135
DICER1	~0 (perfect separation)	–	0.999
Others	0.83	0.22–3.08	0.781
**RAI (yes vs no)**	0.32	0.14–0.77	0.011

Table S6. Age-stratified predictions

Predicted non-excellent (Indeterminate + Biochemical + Structural), %

**Age → / Mutation ↓**	**Wild-type**	***BRAF V600E***	***RET fusion***	***NTRK1/3***	***RAS***	***DICER1***	**Others**
**0–8**	42.6	71.4	70.8	**79.3**	22.4	**0.0**	59.3
**9–14**	32.2	61.5	60.8	**71.1**	15.6	**0.0**	48.2
**15–18**	22.2	49.0	48.3	**59.7**	10.0	**0.0**	35.9
**19–25**	15.7	38.5	37.8	**49.1**	6.7	**0.0**	26.8

Predictions in cells with zero observed mutation-positive cases are model-based extrapolations (e.g., BRAF V600E at ≤8 years) and may be unstable

Table S7. Unpenalized (MLE) logistic regression-Adjusted odds ratios for Non-excellent vs Excellent (reference = Wild-type)

Mutation group	aOR (non-excellent)	95% CI	p-value
BRAF V600E	**3.61**	1.34-9.69	**0.011**
RET fusion	**3.36**	1.07-10.56	**0.038**
NTRK1/3	**3.76**	1.22-11.61	**0.021**
RAS (N/H/K)	0.36	0.04-3.33	0.366
DICER1	~0 (perfect separation)	-	0.999
Others	1.51	0.48-4.72	0.479

Standard maximum-likelihood logistic regression; DICER1 shows perfect separation (0 Non-excellent), so the MLE aOR collapses toward 0 with no finite CI.

Table S8 . Disease Status at Last Clinical Evaluation X Age group

Age group	Excellent	Indeterminate	Biochemical Incomplete	Structural Incomplete	Total (n)
0-8	2 (50.0%)	0 (0.0%)	0 (0.0%)	2 (50.0%)	4
9-14	38 (56.7%)	4 (6.0%)	11 (16.4%)	14 (20.9%)	67
15-18	54 (64.3%)	7 (8.3%)	18 (21.4%)	5 (6.0%)	84
19-25	26 (81.2%)	1 (3.1%)	3 (9.4%)	2 (6.2%)	32

Because 43.8% of cells had an expected count less than 0.5, the Pearson χ^2 test was not valid. Therefore, the Fisher–Freeman–Halton exact test was used ($\chi^2 = 15.83$; $p = 0.044$).

Table S9 . Disease Status at Last Clinical Evaluation X Final Pathology

Histopathology	Excellent	Indeterminate	Biochemical Incomplete	Structural Incomplete	Total (n)
Benign	7 (100.0%)	0 (0.0%)	0 (0.0%)	0 (0.0%)	7
Low-Risk Neoplasm	1 (100.0%)	0 (0.0%)	0 (0.0%)	0 (0.0%)	1
Malignant	112 (62.6%)	12 (6.7%)	32 (17.9%)	23 (12.8%)	179

Because 66.7% of cells had an expected count less than 0.5, the Pearson χ^2 test was not valid. Therefore, the Fisher–Freeman–Halton exact test was used ($\chi^2 = 4.64$; $p = 0.665$).

Table S10. Disease Status at Last Clinical Evaluation X Bethesda Category

Bethesda (3rd ed.)	Excellent	Indeterminate	Biochemical Incomplete	Structural Incomplete	Total (n)
I (ND)	2 (100.0%)	0 (0.0%)	0 (0.0%)	0 (0.0%)	2
II (Benign)	12 (100.0%)	0 (0.0%)	0 (0.0%)	0 (0.0%)	12
III (AUS)	11 (78.6%)	1 (7.1%)	2 (14.3%)	0 (0.0%)	14
IV (FN)	17 (68.0%)	3 (12.0%)	4 (16.0%)	1 (4.0%)	25
V (SFM)	17 (77.3%)	2 (9.1%)	2 (9.1%)	1 (4.5%)	22
VI (Malignant)	57 (55.9%)	5 (4.9%)	23 (22.5%)	17 (16.7%)	102

Because 66.7% of cells had an expected count less than 0.5, the Pearson χ^2 test was not valid. Therefore, the Fisher–Freeman–Halton exact test was used ($\chi^2 = 18.08$; $p = 0.164$).

Table S11. "Excellent" vs "Non-excellent" (unadjusted odds vs Wild-type)

(Non-excellent = Biochemical Incomplete + Structural Incomplete + Indeterminate; Haldane–Anscombe correction used for zero cells)

Group	Non-excellent	Excellent	OR vs Wild-type	95% CI
BRAF V600E	19	18	**3.96**	1.60–9.80
RET fusion	13	14	**3.48**	1.30–9.35
NTRK1/3	13	9	**5.42**	1.87–15.67
**Others**	9	19	1.78	0.64–4.91
RAS	1	8	0.47	0.05–4.12
DICER1	0	7	0.24	0.01–4.55

Table S12. Detailed characteristics of the seven patients with final benign histology and available somatic testing.

Case	Age group	Sex	Benign subtype code	Driver group
1	9–14	2	Thyroid follicular nodular disease	DICER1
2	19–25	2	other	Others
3	9–14	1	Follicular thyroid adenoma	Others
4	15–18	2	other	Wild-type
5	15–18	2	other	Others
6	19–25	2	Follicular thyroid adenoma	DICER1
7	19–25	2	Follicular thyroid adenoma	Wild-type
